# Increased cerebellar gray matter volume in head chefs

**DOI:** 10.1371/journal.pone.0171457

**Published:** 2017-02-09

**Authors:** Antonio Cerasa, Alessia Sarica, Iolanda Martino, Carmelo Fabbricatore, Francesco Tomaiuolo, Federico Rocca, Manuela Caracciolo, Aldo Quattrone

**Affiliations:** 1 Istituto di Bioimmagini e Fisiologia Molecolare, Consiglio Nazionale delle Ricerche, Catanzaro, Italy; 2 Istituto Istruzione Superiore “Mancini”, Cosenza, Italy; 3 Federazione Italiana Cuochi, Rome, Italy; 4 Fondazione Volterra Ricerche “Auxilium Vitae”, Volterra, Italy; 5 Unità Operativa di Psicologia Clinica, Azienda Ospedaliero Universitaria, Pisa, Italy; 6 Istituto di Neurologia, Università "Magna Graecia", Catanzaro, Italy; Tokyo Medical and Dental University, JAPAN

## Abstract

**Objective:**

Chefs exert expert motor and cognitive performances on a daily basis. Neuroimaging has clearly shown that that long-term skill learning (i.e., athletes, musicians, chess player or sommeliers) induces plastic changes in the brain thus enabling tasks to be performed faster and more accurately. How a chef's expertise is embodied in a specific neural network has never been investigated.

**Methods:**

Eleven Italian head chefs with long-term brigade management expertise and 11 demographically-/ psychologically- matched non-experts underwent morphological evaluations.

**Results:**

Voxel-based analysis performed with SUIT, as well as, automated volumetric measurement assessed with Freesurfer, revealed increased gray matter volume in the cerebellum in chefs compared to non-experts. The most significant changes were detected in the anterior vermis and the posterior cerebellar lobule. The magnitude of the brigade staff and the higher performance in the Tower of London test correlated with these specific gray matter increases, respectively.

**Conclusions:**

We found that chefs are characterized by an anatomical variability involving the cerebellum. This confirms the role of this region in the development of similar expert brains characterized by learning dexterous skills, such as pianists, rock climbers and basketball players. However, the nature of the cellular events underlying the detected morphological differences remains an open question.

## Introduction

It is commonly believed that “cooking skills” involve simple routine or recreational activities without attracting a real scientific interest. Recent evidence from neurorehabilitation studies changed the way these kinds of skills are viewed. Indeed, cooking tasks created with a computer-based framework provided a scientifically grounded way to improve executive functions in stroke patients or to stave off cognitive decline in patients with Alzheimer’s disease [1,2]. A critical factor in the success of this kind of cognitive intervention was the high acceptability, satisfaction and motivation stemming from the idea of cooking a meal.

What does a “cooking ability” actually mean? First of all, cooking involves several executive functions, such as: inhibitory control, retrieval of information, mental shifting (switching back and forth between tasks and mental sets) and sustained attention. More importantly, cooking means planning: scheduling when to start and stop cooking each food, and deciding on the priority of dishes and continuously updating contextual information (foods that are near to start or end of their cooking times require closer monitoring or attention than other foods) [3–5]. Apart from executive functions, chef-related daily activity basically requires learning new motor schemes as well as dexterous bimanual coordination in order to maximize the food’s simultaneous completion, thus ensuring quality [6, 7].

Obviously, there is a difference between how this specific ability is carried out by a non-expert in a domestic setting in comparison with an expert, that is, people who have devoted a large amount of their life to this practice: chefs. This word is derived from the term *chef de cuisine*, the head of a kitchen [6]. The French word comes from Latin *caput* closely related to the English "*chief*". To obtain the title—head chef,- a professional cooking skill must have been attained through long-term experience and have full authority in the brigade management of a restaurant [7].

The process that makes people faster, more accurately and efficiently is also called skill learning. To achieve this, the brain needs to change, establishing new stable connections between neural populations with the resulting increased responsiveness of specific brain areas. Whether “cooking skills” are embodied in a specific neural circuitry has never been investigated, although four substantial pieces of evidence would seem to sustain this hypothesis.

First, as stated by Neumman et al., [8] *“expert performance constitutes the endpoint of skill acquisition and is accompanied by widespread neuroplastic changes”*. Neurogenesis, microglia proliferation, synaptogenesis, angiogenesis and axonal sprouting are all neurobiological processes that may occur in response to learning and experience [9,10,11]. Generally, “experts” are people who have had many years of training in a specific domain [8]. Bearing in mind that a chef becomes director of a kitchen (head chef) after several years of experience, it could be included in the category of the expert brain.
Second, the vast majority of brain functions characterizing the chef*-*related daily activity is basically controlled by the cerebellum [12]. Generally, the anterior lobe (lobules from I to V) is especially involved in motor execution, synchronization, coordination, control and prediction, whereas the posterior lobe (lobules from VI, VII including Crus I-II) is involved in executive functions, language and attention/spatial processing [12,13,14].
Third, the cerebellum is one of the principal brain regions involved in motor /cognitive learning [14]. Indeed, animal studies have demonstrated that process of acquiring new repertoires of movements and skills to perform them through practice is specifically embodied in the cerebellum, and this produces morphological plasticity [15–19].
Finally, several neuroimaging studies in humans have also confirmed that expertise-dependent plasticity occurs in the cerebellum after long-term motor and cognitive training, and that the amount of practice is an important factor influencing the extent of anatomical reorganization [20–22]. The majority of findings in this field of study have relied on musicians who have served as a model for studying how expertise can change the neuroanatomy in humans [11, 20]. However, not only musicians demonstrate these kinds of neurobiological features. Indeed, there are other similar features in other career groups, such as world-class mountain climbers [22] and basketball players [23] who have shown specific gray matter (GM) increases in the cerebellar cortex. Neuroanatomical changes strongly related to expertise have also been detected in other groups where the individual level of proficiency is difficult to establish, such as typists [24] and taxi drivers [25].

Following this large amount of evidence, we hypothesize that, in agreement with findings already demonstrated in other highly dexterous motor/cognitive expertise domains [22,23],chefs could be characterized by neural reorganization above all involving the cerebellum. To test this hypothesis, we combined two distinct morphologic neuroimaging measurements widely employed in expertise-related studies [21–23, 25]: voxel-based morphometry (VBM) [21–23, 25–26] and automated volumetry (Freesurfer) [27, 28] in a multimethod unbiased approach [29]. We used both methods because they provide complementary information that cannot be obtained by using one method alone. VBM performs a statistical mapping of differences between groups in brain morphology voxel-by-voxel, returning a GM “attenuation” or “concentration” measure. On the other hand, automatic volumetry performed by Freesurfer is a quantitative measurement of specific brain regions, which has been validated in several psychiatric and neurological domains [30]. We therefore aim to demonstrate the potential of multimodal assessment by converging results from morphological magnetic resonance imaging (MRI).

## Materials and methods

### Participants

Eleven right-handed head chefs with more than five years brigade management expertise were recruited from a list of professional restaurants provided by the Federation of Italian chefs in Calabria. All chefs have obtained at least one national prize/award for their ability. The mean of years as a head chef was: 19.8 ± 10.3. In the last few years three of 11 became executive chefs after 35 years as head chefs. Overall, considering the last period (5 years), chefs reported an average size of brigade staff: 8.8 ± 3.5, whereas sous-chefs ranged from 1 to 2 and number of hours per day in the kitchen was 16 ± 2.1. Three of 11 chefs hold one Michelin star.

Chefs were compared with 11 age-/ sex-/ and educational level-matched right-handed volunteers without specific cooking abilities. These individuals were recruited by our large neuroimaging database [31] and the vast majority was student or office worker. Experts and non-experts individuals were individually pair-matched by a computer-generated program, according to their age and sex.

Exclusion criteria were: (i) major medical illnesses and/or known or suspected history of mental disorders (i.e. schizophrenia, mood, anxiety, personality and/or any other significant mental disorders), alcoholism or drug dependency and abuse (i.e., antidepressant or psychoactive drugs), (ii) presence of vascular brain lesions, brain tumor and/or marked cortical and/or subcortical atrophy and (iii) no excessive movement artifacts during MRI acquisition. Participants were not medicated and in good health. Six chefs and five non-experts individuals were smokers. Before MRI exams, enrolled subjects filled out personality inventory and underwent neuropsychological assessment (see below). All participants gave written informed consent to participate in the present study, approved by the Ethical Committee of the University ‘Magna Graecia’ of Catanzaro according to the declaration of Helsinki.

### Neuropsychological assessment

All individuals enrolled for the MRI experiment completed an extensive series of neuropsychological tests, which were administered by an experienced clinical neuropsychologist (I.M.) blind to any other result. The following cognitive functions were evaluated:

1. executive control; Tower of London (TOL) and Stroop task (ST). The TOL is a test for evaluating motor/cognitive planning abilities consisting in moving colored balls within a limited number of moves in order to achieve a given goal configuration. The ST evaluates interference resolution and cognitive inhibition when the subject has to identifying the ink of a color word, which is incongruent to the meaning of the presented color.
2. Working memory/Attention were assessed using: a) Paced Auditory Serial Addition Test (PASAT)-2’; b) Trial Making Test A-B (TMT); c) Symbol Digit Modality Test (SDMT) and d) Digit Span Forward and Backward. The PASAT and DIGIT SPAN are measures of working memory and speed of information processing, whereas SDMT assesses key neurocognitive functions that underlie many substitution tasks, including attention, visual scanning and motor speed. Finally, TMT consists of two parts (A & B) in which the subject is instructed to connect a set of 25 dots as quickly as possible while still maintaining accuracy. This is employed to assess visual/attention switching ability.
3. Short- and long-term verbal/spatial memory were measures using the Rey Auditory Verbal Learning Test (RAVLT) and the Rey Osterrieth Complex Figure (ROCF). In the first, participants are given a list of 15 unrelated words repeated over five different trials and are asked to repeat. Another list of 15 unrelated words are given and the subject must again repeat the original list of 15 words and then again after 30 minutes. In the visual version (ROCF), it is asked to reproduce a complicated line drawing, first by copying it freehand (recognition), and then drawing from memory (recall).
4. Verbal ability was assessed with Word List Generation (WLG) test, which explores verbal fluency on semantic stimulus by asking the subject to produce as many words as possible belonging to a semantic category.

### Personality assessment

To better characterize the chefs realm, individuals underwent psychological assessment with the Italian version of the revised NEO-personality-inventory (NEO-PI-R), which measures 30 facets, six for each of the five major dimensions of personality [32,33]. The NEO-PI-R is one of the most employed questionnaires for assessing the broad five personality dimensions. Raw scores were converted to T scores (M = 50, SD = 10) using combined-sex norms [34].

Since specific personality features have been demonstrated to be associated with anatomical variability of the cerebellum [35], we further assessed the impact of this factor on our morphological data (data in S1 Fig).

### Data acquisition

Scanning was performed on a 3-T scanner (Discovery MR750 General Electric, Milwaukee, Wisconsin, USA) with an 8-channel head coil. We obtained structural MRI data using a 3D T1-weighted spoiled gradient echo (SPGR) sequence (TR: 3.7 ms. TE: 9.2 ms, flip angle 12°, voxelsize 1x1x1 mm’; 368 sagittal slices). Particular care was taken to restrain the subjects’ movements with cushions and adhesive medical tape.

### Voxel-based cerebellar analysis

Cerebellum-optimized voxel-based analysis was performed using the spatially unbiased infra-tentorial template (SUIT) toolbox (http://www.diedrichsenlab.org/imaging/suit.htm)[36]. In the first segmentation step, using the unified segmentation approach [26] as implemented in SPM8 (http://www.fil.ion.ucl.ac.uk/spm/), all brain regions expect for the brainstem and cerebellum were removed. Subsequently, each individual's cerebellar gray and white matter segments were normalized onto the SUIT atlas template, allowing for an improved alignment of individual fissures and cerebellar subregions when compared to conventional whole-brain VBM [36]. A modulation of the segmented gray and white matter probability maps was applied in order to compensate for volume changes during spatial normalization by multiplying each voxel's intensity value with the Jacobian determinants. Before statistical analysis, all probability images were smoothed with a 8 mm full-width at half-maximum (FWHM) smoothing kernel in SPM8.

### Automated cerebellar volumetry

To corroborate voxel-based findings we further performed cerebellar volumetry by using Freesurfer [27, 29, 30, 35], a widely used and freely available automated processing pipeline that quantifies brain anatomy (http://freesurfer.net). Automated labeling and quantification of cerebellar volume was performed using version 5.3 installed on Ubuntu 14.04.1. The automated procedures for volumetric measures of several deep GM structures have been previously described [29, 37]. In brief, this procedure automatically provided segments and labels for up to 40 unique structures and assigned a neuroanatomical label to each voxel in an MRI volume based on probabilistic information estimated automatically from a manually labeled training set. The automated subcortical segmentation performs by Freesurfer requires these steps: first, an optimal linear transform is computed that maximizes the likelihood of the input image, given an atlas constructed from manually labeled images. A non-linear transform is then initialized with the linear one, and the image is allowed to further deform to better match the atlas. Finally, a Bayesian segmentation procedure is performed, and the maximum a posteriori estimate of the labeling is computed. Freesurfer subcortical segmentation showed a high overlap and high volumetric correlations with manual segmentation and high test–retest reliability [30, 37, 38]. In addition to the recommended segmentation check proposed by Freesurfer, an external neuroradiologist, blind to the subject’s identity, checked visually all segmented images to ensure that there was no error in the label process. None apparent segmentation error, especially within the cerebellum, was detected on multiple slices. Subcortical and estimated total intracranial volumes (eTIV) were taken from the *aseg*.*stats* Freesurfer output file. Normalized right and left cerebellar GM and white matter (WM) values were calculated as follows: [raw cerebellar volume/eTIV]*1000 [29, 31, 35, 38]. No significant difference was detected for eTIV values among groups.

### Resting-state functional connectivity analysis

To further evaluate if neuroanatomical changes in the chefs’ brain might also be associated with functional reorganizations, we performed a seed-based resting-state fMRI connectivity analysis. As for morphological data, we used two distinct neuroimaging approaches to evaluate the presence of functional changes. fMRI data did not provide sufficient evidence to empirically support the hypothesis of functional reorganization in the chefs*’* brain (data in S2–S4 Figs).

### Statistical analysis

Statistical analyses for all demographical and cognitive variables were performed with Statistical Version 6.0 (www.statsoft.com). Assumptions for normality were tested for all continuous variables using the Kolmogorov–Smirnov test. All variables were normally distributed except for educational level. Unpaired *t-* and Mann-Whithey *U-*tests were used to assess potential differences between groups. All statistical analyses had two-tailed alpha levels of < 0.05 for defining significance.

For voxel-based analysis, an independent two-sample *t*-test analysis was used to evaluate the difference between chefs and non-experts. Age and eTIV was included in the model as covariates of no-interest. The association between anatomical variability and demographical/cognitive scores characterizing chefs was evaluated using the multiple regression function of SPM8. Voxel-based maps were thresholded using correction for multiple comparisons: family wise error (FWE) < 0.05 [39–41].

Finally, the same statistical approach was used to evaluate differences between groups in cerebellar volumetric measurements as obtained by Freesurfer’s segmentation. Statistical analysis had a two-tailed level of < 0.05 for defining significance.

## Results

### Behavioral correlates of Chef’s expertise

Any significant difference was detected in demographical/psychological variables between groups. Neuropsychological examination did not reveal significant cognitive ability except for the TOL, where chefs showed higher performance with respect to non-expert individuals (*t*_(20)_ = 3.3; p-level = 0.002)(Table 1).

**Table 1:** Demographic and cognitive characteristics.

**Demographical Data**			
**Variables**	**Chefs**	**Non-experts**	***p* values**
**Gender (f/m)**	1/10	1/10	-
**Educational Level (y)**	13 (13–18)	16 (13–18)	0.22^§^
**Age (years)**	39.7± 1.2	41.1±12.1	0.7^װ^
**Neuropsychological Data**			
**RAVLT IR**	14.5±2.8	15.5±4.5	0.56^װ^
**RAVLT DR**	20.9±1.8	21.4±5.2	0.79^װ^
**ROCF IR**	29.6±3.3	33.9±3.9	0.12^װ^
**ROCF DR**	11.6±4.7	14.9±5.1	0.26^װ^
**WLG**	35.5±12.7	40±11.4	0.28^װ^
**SDMT**	52±11.1	61.1±9.7	0.16^װ^
**PASAT 2’**	40.1±8.5	42.5±7	0.51^װ^
**TMT A-B**	65.1±22.3	49±23.1	0.15^װ^
**ST**	25±5.	23.1±4.9	0.23^װ^
**TOL**	30.9±1.7	28.8±2.3	0.002^װ^
**Digit Span Forward**	5.8±1	6.3±0.9	0.17^װ^
**Digit Span Backward**	4.5±0.8	5.4±0.9	0.19^װ^
**Psychological Profile**			
**Neuroticism**	57.3±11.2	53.1±8	0.31^װ^
**Extraversion**	52.4±11.1	51.5±10.2	0.93^װ^
**Openness**	53.2±11.9	53.3±9.3	0.38^װ^
**Agreeableness**	45.3±11.3	47.8±7.9	0.32^װ^
**Conscientiousness**	54.9±11.7	52±11.9	0.32^װ^

Data are given as mean values (SD) or median values (range) when appropriate. RAVLT IR and RAVLT DR: Rey Auditory-Verbal Learning Test Immediate and Delayed Recall; ROCF IR and ROCF DR: Rey Osterrieth Complex Figure Immediate and Delayed Recall; WLG: Word List Generation; SDMT: Simbol Digit Modality Test; PASAT 2’: Paced Auditory Serial Addition Test 2 sec; TMT: Trial Making Test A-B; ST: Stroop task; TOL: Tower of London.

^װ^ = Two-sample t test

^§^ = Mann-Whitney test

### Voxel-based cerebellar analysis

**Fig 1:**
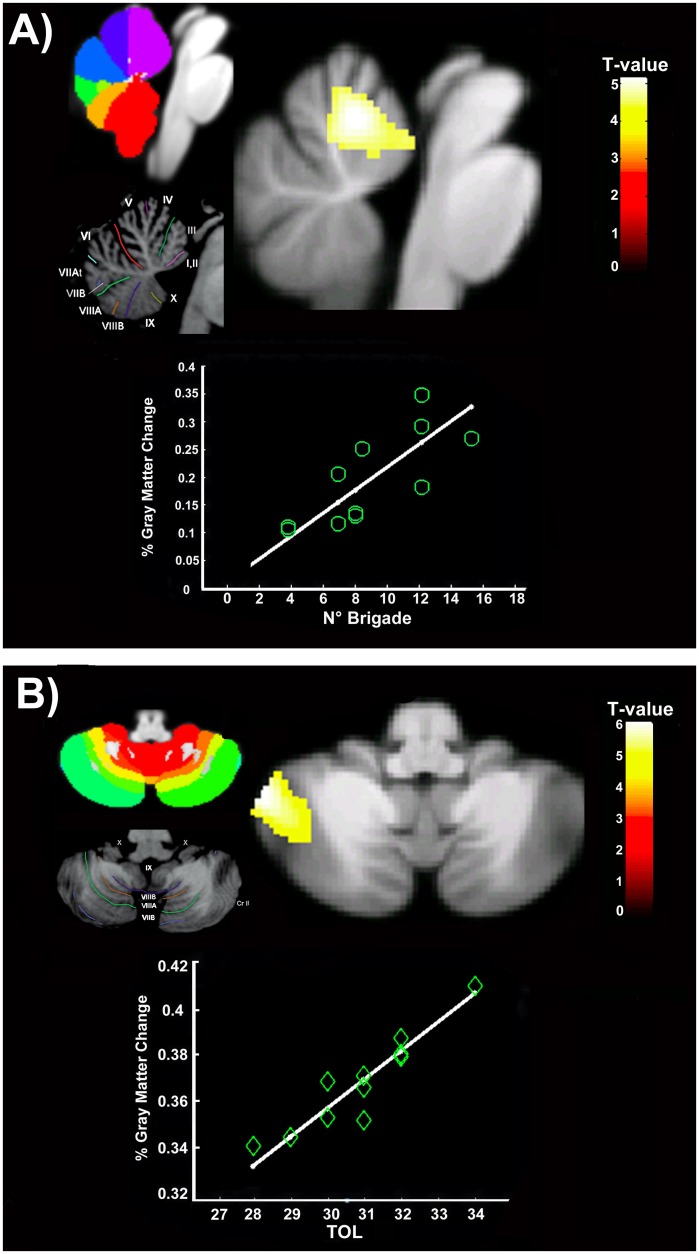
Voxel-based differences between Chefs and non-expert individuals. Statistical maps displaying corrected clusters (FWE < 0.05) of significant gray matter increasing in the anterior cerebellar vermis and the left posterior cerebellar lobule These neural patterns were also significantly associated with specific variables. (A) The increased gray matter density in the anterior vermis was correlated with the magnitude of the brigade staff: more people to synchronize higher neuronal density. (B) A similar linear correlation was detected in the posterior cerebellar lobule where high performance in the TOL test was associated with increased gray matter volume. Statistical maps related to significant effects within the cerebellum have been plotted on the SUIT space. To improve anatomical identification, labeling based on the probabilistic SUIT atlas and Schmahmann’s MRI atlas [63] was showed. The color bar represents *t* statistics. Images are displayed in neurological convention. TOL: Tower of London test.

When compared with non-expert individuals, chefs showed significant enlargement of two specific clusters localized in the anterior vermis (lobules III-IV-V; *t-level = 4*.*9; P*_*FWE*_ *= 0*.*03; cluster (K) = 209; MNI coordinates x*: *-1; y*: *-54; z*: *-14)*(Fig 1A) and in the left posterior cerebellar lobule (Crus II/VIIB; *t-level = 5*.*97; P*_*FWE*_ *= 0*.*005; cluster (K) = 344; MNI coordinates x*: *-51; y*: *-49; z*: *-44)* (Fig 1B) with respect to non-experts.

To evaluate whether the degree of brain enlargement was associated with particular demographical or cognitive variables in the chefs*’* brain, simple regression analysis was performed. The magnitude of the brigade staff positively correlated with the GM volumes of the anterior vermis *(t-level = 6*.*84; P*_*FWE*_ *= 0*.*04; cluster (K) = 35; MNI coordinates x*: *-2; y*: *-55; z*: *-6)*. In other words, the increased number of people in the kitchen who needed to be coordinated and synchronized was associated with increased GM volume in the “motor” cerebellar lobule (Fig 1A). Moreover, performance on the TOL test was positively associated with increased GM volume in the left posterior cerebellar lobule *(t-level = 11*.*5; P*_*FWE*_ *= 0*.*01; cluster (K) = 516; MNI coordinates x*: *-46; y*: *-81; z*: *-32)* (Fig 1B).

### Automated cerebellar volumetry analysis

**Fig 2:**
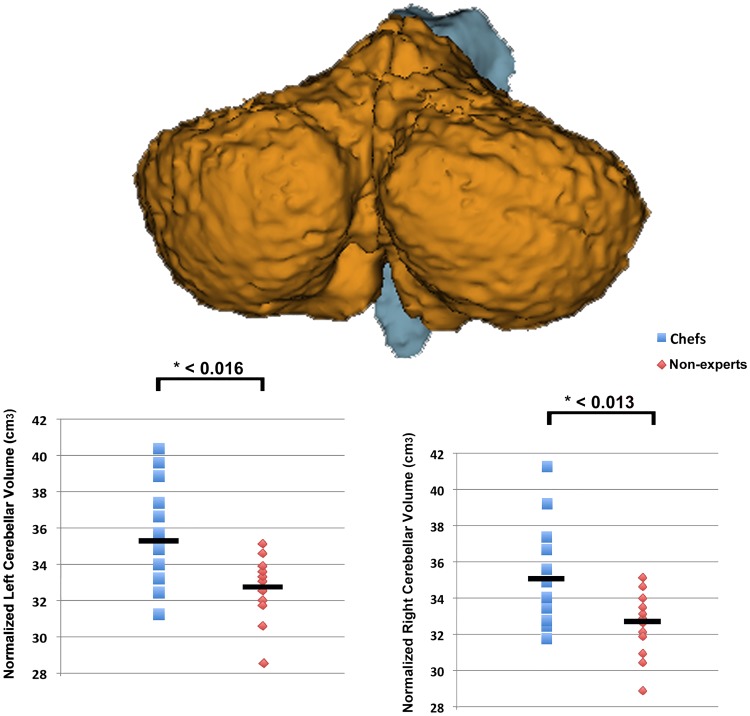
Sample color-coded automated brain segmentation results. A 3D surface image (created with 3D Slicer v 4.6, www.slicer.org) showing typical automated subcortical segmentation of the cerebellum performed by FreeSurfer (v 5.3). Scatter plot of the mean normalized volumes of the left and right cerebellar cortex for each single subject has been plotted. Advanced neuroimaging analysis reveals bilateral cerebellar volumetric increase in the chef group with respect to non-expert individuals.

The VBM-derived findings were supplemented with automated measurements of the cerebellar volumetry as provided by Freesurfer toolbox. Results revealed the expected increased of cerebellar GM in the chef group either for the left (*t*_(20)_ = 7.1; p-level = 0.016; mean ± SD, chefs: 35.4 ± 3.1 cm^3^; non-experts: 32,.9 ± 1.5 cm^3^) or right hemisphere (*t*_(20)_ = 7.6; p-level = 0.013; mean ± SD, chefs: 35.2 ± 3.2 cm^3^; non-experts: 32,6 ± 1.6 cm^3^) with respect to non-expert individuals (Fig 2), whereas no significant differences were detected investigating the WM volume.

## Discussion

The acquisition and practice of skills over the long-term leads to macroscopic brain plasticity both in animal [17] and in humans [8]. This neuroplasticity is considered adaptive since is assumed to be essential for optimizing outputs in order to be faster and more accurate. Using a multi-method structural neuroimaging approach [29, 31, 38] we demonstrated, for the first time, that a specific career group, head chefs, are characterized by anatomical variability in the cerebellum, a region strongly involved in the development of skill learning and expertise [8, 10–12,18].

The first cluster identified by voxel-based approach is within the anterior vermis. Basically, it is well-known that performance improvements in a variety of motor learning paradigms (from simply tying knots to dexterous skills such as playing musical instruments) induce evident neural changes, mainly including the anterior cerebellar lobule [18]. The translation of learned motor skills into fast and accurate procedural motor patterns is achieved through the generation of internal models of body movements embodied in the cerebellum [42–46]. Internal models are neural mechanisms that mimic the input-output properties of motor acts or thoughts. Computationally speaking, the cerebellum generates an estimate (spike train) of the true state of the peripheral motor system, integrating incoming sensory information with predictions of the consequences of outgoing motor commands [41]. If a match is obtained, then it is assumed that the next incoming event will be more quickly predicted by the internal models [41–47]. In line with its role in the “state estimation” control of motor skills, neural reorganization in the anterior vermis has also been observed in other expert groups, such as world-class mountain climbers [22], basketball players [23] and pianists [20]. In agreement with other studies in this field [8,10, 11], we hypothesize that the volumetric enlargement detected in the chefs*’* brain may represent neuroplasticity caused by years of daily training and exercise, although we recognize the lack of a direct behavioral measure of this expertise. On the other hand, the detected pattern of neural change was found to be associated with a specific chefs-related skill: the magnitude of the brigade staff. Indeed, a *head* chef must have full authority in the brigade management of a restaurant [7], that is, they need to accurately and efficiently coordinate, synchronize and predict events in their kitchen [6, 7]. We hypothesize that the estimation processing of body movements might extend beyond the peripersonal body space, including the distal action control of other people. This might explain the significant association between the increased volumetry of the anterior cerebellum with the brigade magnitude (Fig 1). However, monitoring and organizing the tasks of a variety of other employees would seem to especially belong to the cognitive rather than the motor realm. This is not surprising since previous evidence has demonstrated that the anterior vermis is also specifically implicated in cognitive aspects of sensorimotor task performance and learning [48, 49]. However, without the employment of a useful fMRI-related motor evaluation or behavioral marker to discriminate chefs*’* expertise into motor skills, the proposed interplay between cerebellar involvement and internal models generation in chefs remains speculative.

On the other hand, the high performance during the TOL provides an interesting cognitive hallmark of chef-related expertise. TOL is extensively used for assessing planning ability [50]. This cognitive skill is strongly required during daily work activities, where chefs are continuously stressed by scheduling, predicting and planning a vast series of distal or abstract consequences of actions, mentally rehearsing entire food preparation sequences, imagining novel ones, whilst simultaneously monitoring and evaluating other people’s activities. At a neurophysiological level, to be performed, TOL mainly requires the involvement of a specific “cognitive” pathway including the dorsolateral prefrontal cortex, superior parietal lobule and posterior cerebellum [51, 52]. The Crus II (as well as Crus I and VIIb lobules), are equally involved during TOL execution [53,54]. Generally speaking, fMRI studies have demonstrated that shorter planning time and fewer moves to complete a TOL sequence require the overactivity of the left Crus I/II lobules [52, 54], which are equally sustained by tight anatomical connections with the prefrontal cortex [51]. Thus, the fact that chefs with a high TOL performance are those with a larger posterior cerebellar volume would seem to be in agreement with previous evidence. With respect to other studies on experts’ brains (chess [55], and mental arithmetic [56]), where significant neural plasticity was detected in domain-specific structures related to the task performed (i.e., visual systems for visuo-spatial experts)[8], the detected neural reorganization of the posterior “cognitive” cerebellum might also be explained by the involvement of internal models. Indeed, the cerebellum is not only involved in synchronizing and controlling motor acts but also contributes to automatizing cognitive functions [12, 57]. When human subjects acquired and repeatedly used cognitive skills (so-called “automation of thought processes” [58]), the major cerebellar lobules involved were the Crus I-II [12].

Some limitations should be mentioned. Firstly, we did not assess the degree of expertise using, for instance, the well-known metric of Michelin stars. There is considerable variability among top experts regarding the level of skill they achieve. This paper also does not consider creativity. A great chef should be very creative and always willing to try something new. Creativity inspires the food’s presentation, which is very important in the overall dining experience. Neuroscience has recently addressed this topic given the difficulty in creating psychometric measures of creativity. Overall, initial evidence highlights the occipito-parietal activity related to creativity [59]. Secondly, the relatively small sample size as well as the unbalanced gender distribution in the chef group may have masked subtle characteristics. Overall, it remains to be established what exactly a chef ‘s expertise entails. Whether the main characteristic is the cooking ability, an additional control group of line-cooks with similar cooking expertise but without the brigade management experience should be considered in order to evaluate to what degree the cerebellum is involved in chef*-*related activity. Finally, the cross-sectional design of our neuroimaging study does not consider the popular conundrum. “Which Came First—the Chicken or the Egg?” In other words, could anatomical differences, detected in chefs with respect to non-experts, have existed before the training experience? Foster and Zatorre [60] suggested that when neural changes are detected in regions that are sensitive and directly involved with expertise-related activity, the predisposition hypothesis is less likely. Overall, although all these issues might represent limitations of the present study, they should be considered as “food for thought” in stimulating future research. One important consequence of our study might be to translate it into a neurorehabilitation realm. Cooking training has been shown to lead to improvements in the efficiency of executive control processing when applied in the elderly [3]. This kind of intervention could be extended, since it could lead to an improvement in rehabilitation programs targeting cerebellar symptoms in multiple sclerosis [61] as well as executive dysfunctions in Parkinson’s disease patients [62].

## Conclusions

This multimodal structural neuroimaging work is aimed at understanding whether expertise in chefs is embodied in specific neural networks. Although the level of proficiency is difficult to establish in this particular career group, our head chefs with long-term practical and business experience, showed specific structural differences within the cerebellum with respect to demographically-matched non-expert individuals. In agreement with the large amount of experience-dependent cerebellar changes described in other experts’ brains (pianists, rock climbers and basketball players [20,22,23]), we hypothesize a similar mechanism for explaining the brain morphological variability depicted in chefs, although the cross-sectional design of this study may only suggest a proof-of-concept for neural plasticity.

## Supporting information

S1 Fig Whole-brain VBM results. 3D/2D surface renders show the significant cluster deriving from the comparison between Chefs with non-expert individuals. Increased gray matter volume in the left primary somatosensory cortex was detected. In the 2D surface red line indicates the precentral sulcus.
(TIF)

S2 Fig Motion artifacts analysis. Plots of the seven mean motion head parameters during resting-state fMRI session for each single expert and non-expert individuals. Figure shows trend of head motion separately for translation (x, y and z direction, first row) and for rotation (pitch, roll and yaw, second row). In the lower part of the figure we show calculation of the Euclidian distance traveled by each subject’s head from the first to the last scan. No significant motion difference was detected during fMRI measurement.
(TIF)

S3 Fig Seed-based functional connectivity analysis by MELODIC/FSL toolbox. The comparison between Chefs and non-expert showed increased communication between the seed placed on the anterior cerebellar lobule and the bilateral secondary somatosensory cortex together with the medial premotor cortex. Considering the second seed placed on the posterior cerebellar lobule, Chefs showed decreased connectivity with right anterior prefrontal cortex.
(TIF)

S4 Fig Seed-based functional connectivity analysis by CONN toolbox. The comparison between Chefs and non-expert showed increased communication between the seed placed on the anterior cerebellar lobule (left panel) and the right motor and premotor cortices (red blob in right panel), although without reaching significant threshold.
(TIF)

S1 File Complementary results on VBM and resting-state functional connectivity data. (DOCX)
