# Supplementary figures and images for: Increased cerebellar gray matter volume in head chefs
Source: PLoS One. 2017 Feb 9;12(2):e0171457. doi: 10.1371/journal.pone.0171457 (PMC5300254; doi:10.1371/journal.pone.0171457)

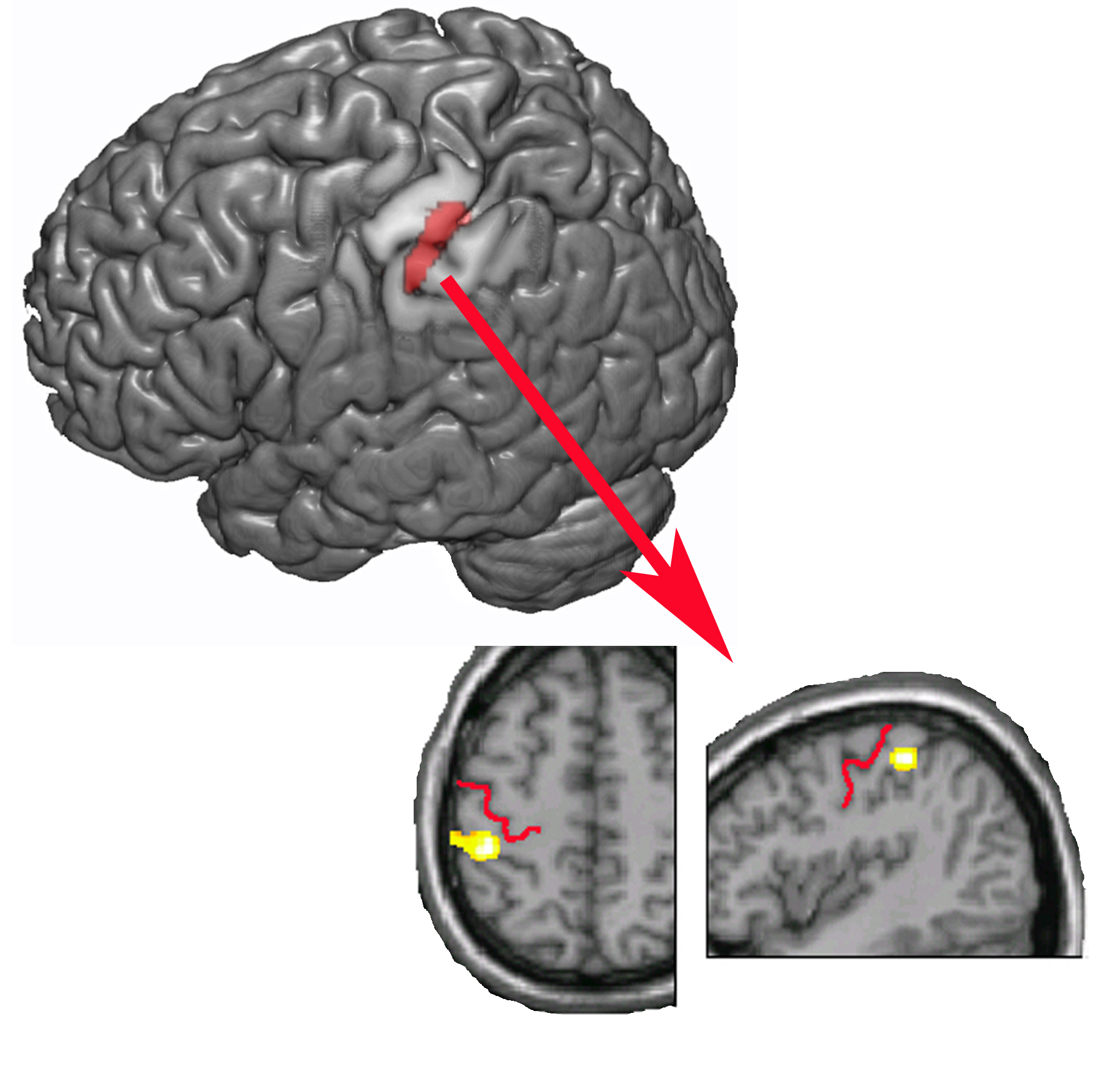

Supplement: S1 Fig — 3D/2D surface renders show the significant cluster deriving from the comparison between Chefs with non-expert individuals. Increased gray matter volume in the left primary somatosensory cortex was detected. In the 2D surface red line indicates the precentral sulcus. (TIF) [file pone.0171457.s001.tif]

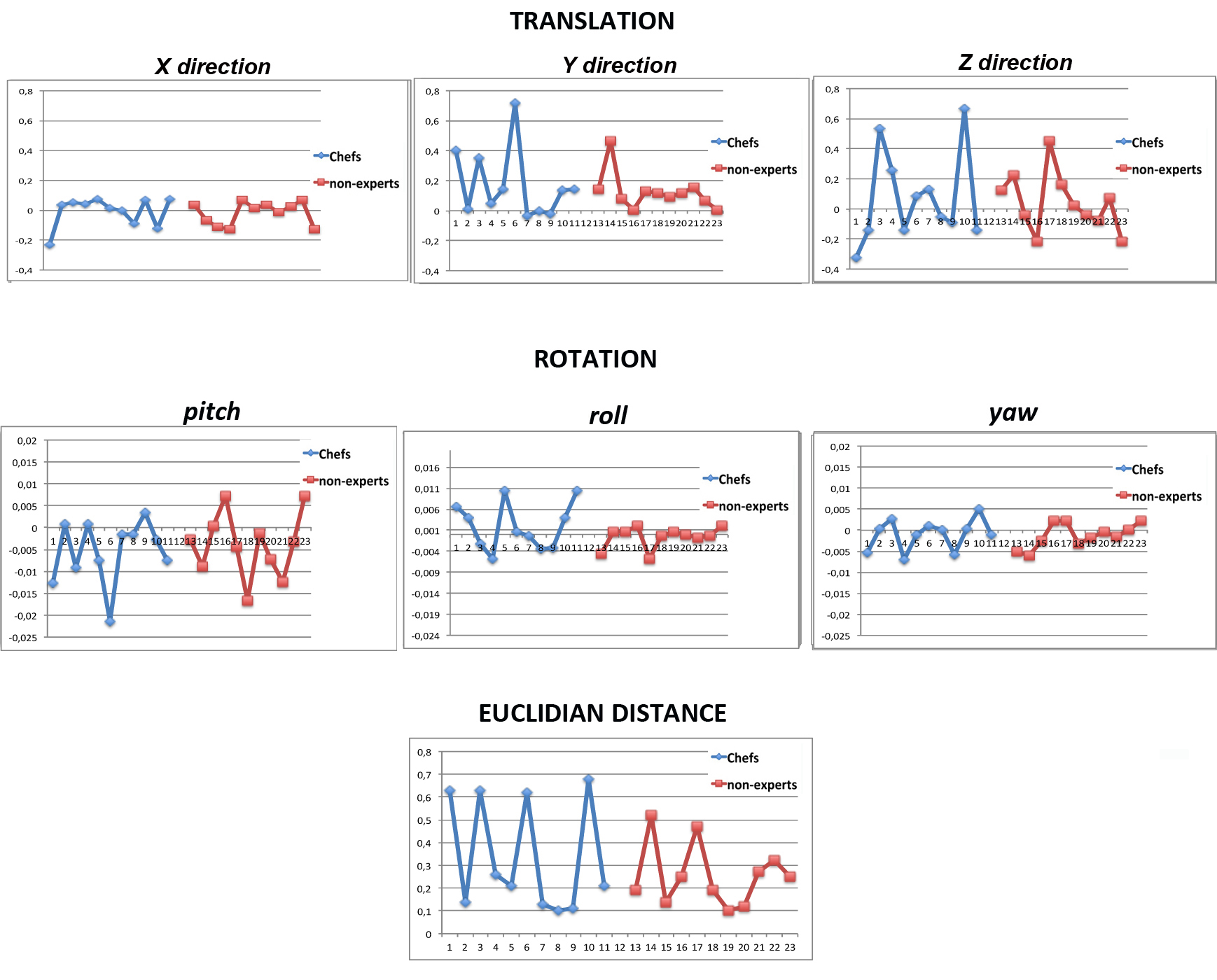

Supplement: S2 Fig — Plots of the seven mean motion head parameters during resting-state fMRI session for each single expert and non-expert individuals. Figure shows trend of head motion separately for translation (x, y and z direction, first row) and for rotation (pitch, roll and yaw, second row). In the lower part of the figure we show calculation of the Euclidian distance traveled by each subject’s head from the first to the last scan. No significant motion difference was detected during fMRI measurement. (TIF) [file pone.0171457.s002.tif]

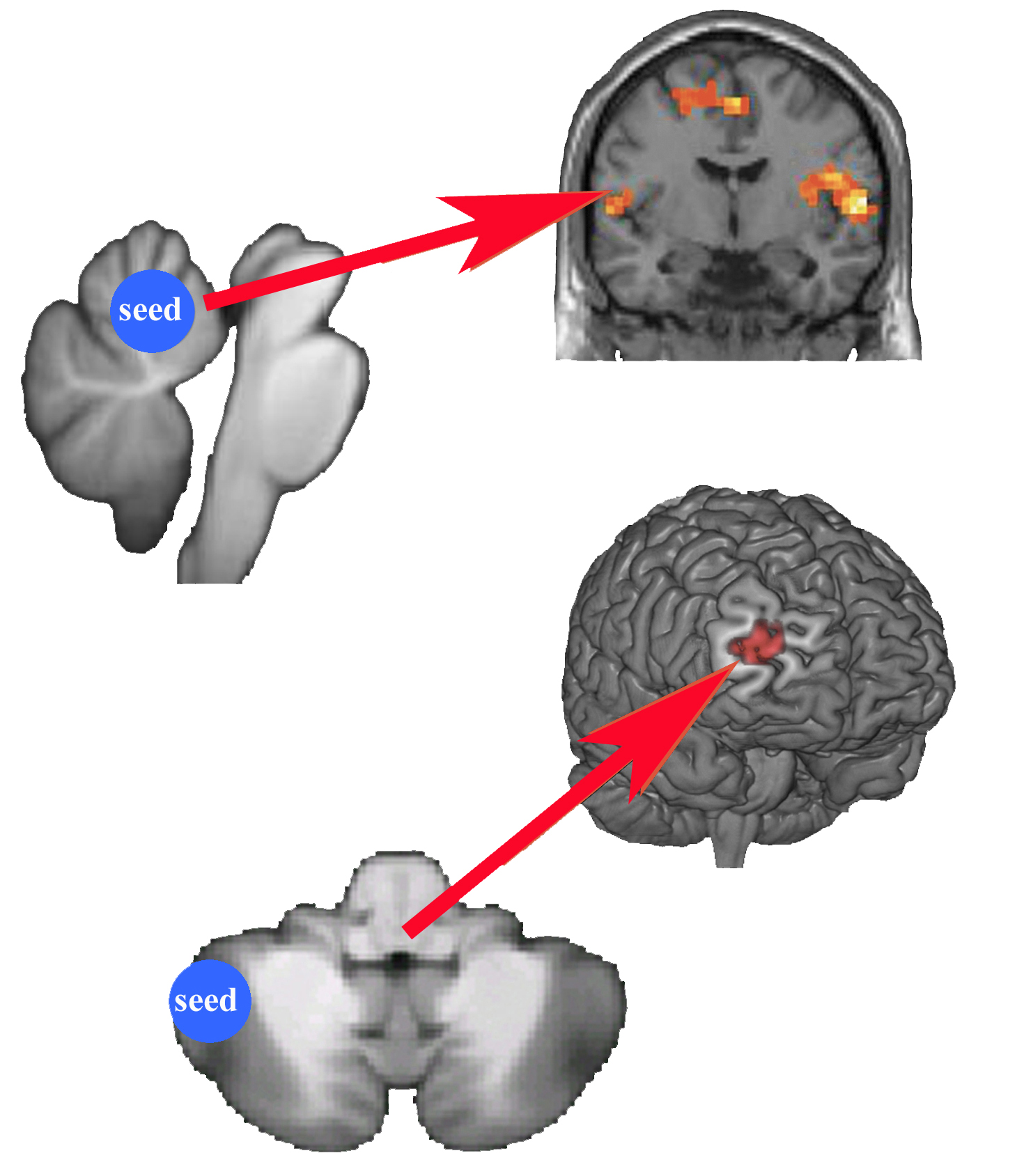

Supplement: S3 Fig — The comparison between Chefs and non-expert showed increased communication between the seed placed on the anterior cerebellar lobule and the bilateral secondary somatosensory cortex together with the medial premotor cortex. Considering the second seed placed on the posterior cerebellar lobule, Chefs showed decreased connectivity with right anterior prefrontal cortex. (TIF) [file pone.0171457.s003.tif]

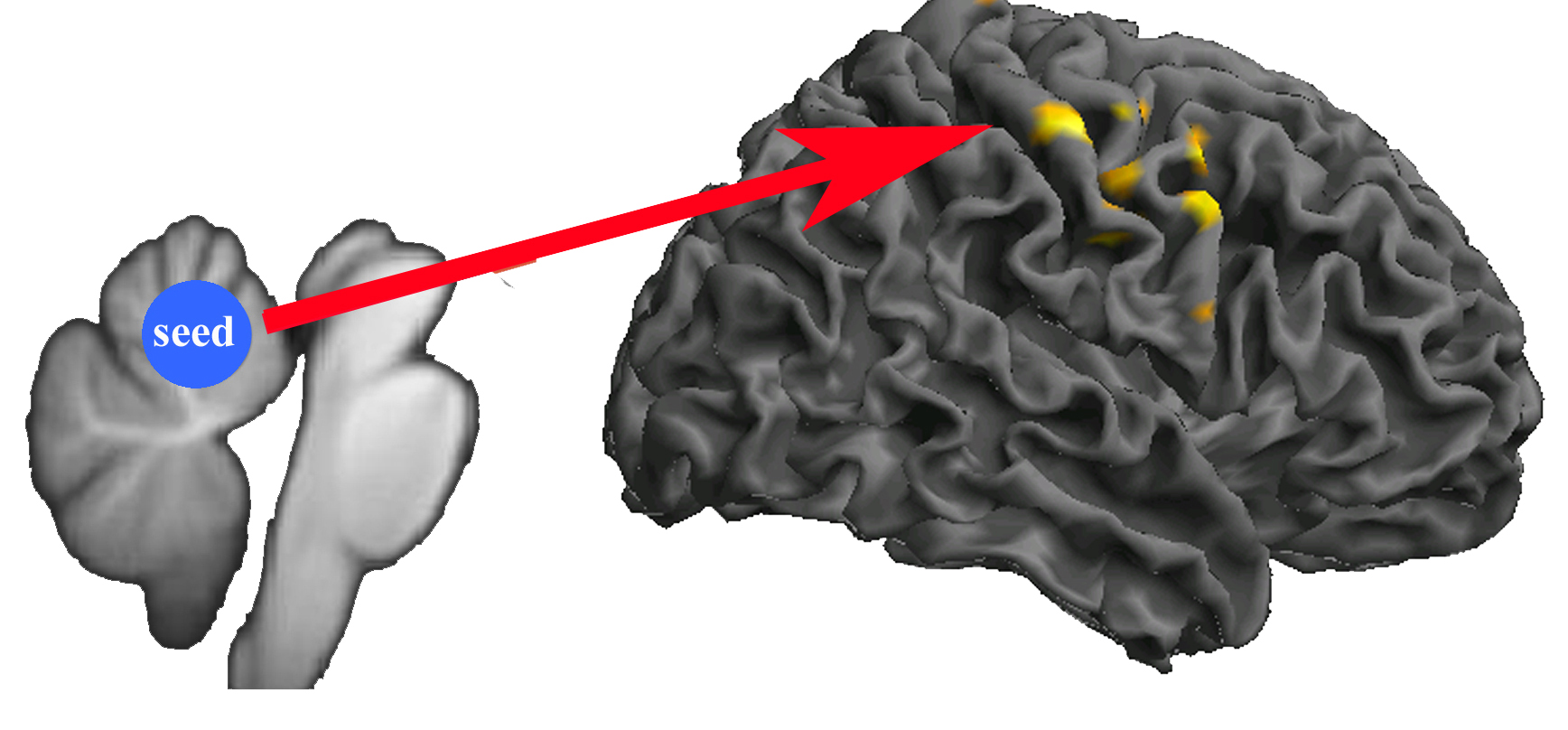

Supplement: S4 Fig — The comparison between Chefs and non-expert showed increased communication between the seed placed on the anterior cerebellar lobule (left panel) and the right motor and premotor cortices (red blob in right panel), although without reaching significant threshold. (TIF) [file pone.0171457.s004.tif]
