# Supplementary material for: Increased cerebellar gray matter volume in head chefs
Source: PLoS One. 2017 Feb 9;12(2):e0171457. doi: 10.1371/journal.pone.0171457 (PMC5300254; doi:10.1371/journal.pone.0171457)
Supplement: S1 File — (DOCX) [file pone.0171457.s005.docx]

**Supplementary Materials for**

**Increased cerebellar gray matter volume in head Chefs**

Antonio Cerasa, Alessia Sarica, Iolanda Martino, Carmelo Fabbricatore, Francesco Tomaiuolo, Federico Rocca, Manuela Caracciolo, Aldo Quattrone

correspondence to: [antonio.cerasa76@gmail.com](mailto:a.cerasa@unicz.it)

**VBM analysis**

**Whole-brain analysis: Methods**

For voxel-based analysis of the entire brain we used the VBM8 toolbox (<http://dbm.neuro.uni-jena.de/vbm.html>). Images were bias-corrected, tissue classified and registered using linear and non-linear transformations, within a unified model [1]. Subsequently, the warped GM segments were affine transformed into Montreal Neurological Institute (MNI) space and were scaled by the Jacobian determinants of the deformations (modulated GM volumes). Finally, the modulated volumes were smoothed with a Gaussian kernel of 8 mm.

The general linear model (GLM) based on Gaussian random field theory statistically analyzed the GM volume maps. An independent two-sample *t*-test analysis detected differences between *Chefs* and non-experts including age and total intracranial volume (TIV) in the model as covariates of no-interest.

Given the lack of literature data linking brain features in *Chefs,* whole-brain analysis was presented by using a less-stringent uncorrected threshold (P_uncorrected_ < 0.001, cluster (k) threshold > 10 voxels).

**Whole-brain analysis: Results**

The assessment of GM changes in the *Chefs’* brain outside the cerebellum revealed the presence of one only cluster detected in the left primary somatosensory cortex (*t-level= 5.19; cluster (K)= 124; MNI coordinates x: -42; y: -34; z: 54)* (Figure S1). Anatomical localization encompasses the homunculus of the hand, thus suggesting that *Chefs* have developed a greater neural representation of sensorial information.

Despite the small sample size and the low statistical threshold, we retain that this pattern of increased GM volume deserves future investigations.


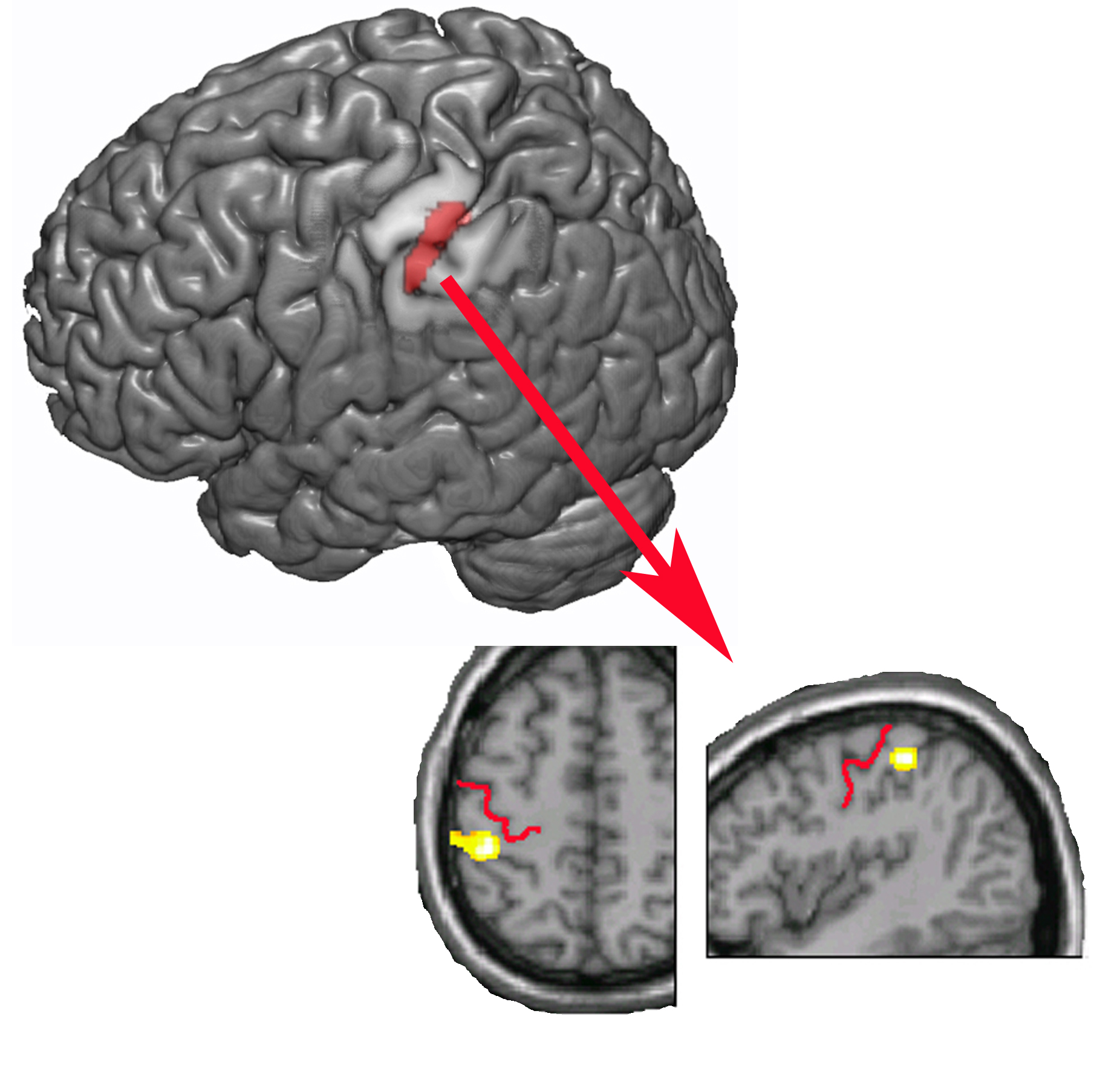


**S1 Fig:** **Whole-brain VBM results.** 3D/2D surface renders show the significant cluster deriving from the comparison between Chefs with non-expert individuals. Increased gray matter volume in the left primary somatosensory cortex was detected. In the 2D surface red line indicates the precentral sulcus.

As showed in the main document (Table 1), *Chefs* tend towards high levels of emotional instability (neuroticism), although without reaching significant threshold. However, despite this lack of behavioral evidence, we performed a further morphological analysis for excluding nuisance variables from our study. Since it has been demonstrated that personality traits might affect anatomical variability, including the cerebellum [2], we re-run VBM analysis including neuroticism scores as nuisance variable. We did not reveal any relevant change in the overall morphological patterns.

Moreover, we tried to directly correlate neuroticism scores with morphological data to evaluate if *Chefs* with high scores are characterized by pre-existent anatomical variability. No significant finding was detected. This evidence indirectly confirmed our previous study [3] demonstrating that anxiety scores are strongly associated with anatomical variability of the limbic system, but only when this personality trait is evaluated with the Hamilton test for anxiety (HAMA) rather than other scales which not strictly evaluated anxiety *per se*.

**Resting-state functional connectivity analysis**

To evaluate whether the detected pattern of cerebellar structural changes was also associated with functional reorganization, we performed a seed-based resting-state functional connectivity analysis. As for morphological analyses, we employed two distinct fMRI approaches: a) MELODIC/FSL toolbox [4,5] (<http://www.fmrib.ox.ac.uk/fsl>); b) COON toolbox [6] (<http://www.nitrc.org/projects/conn)>. We used both methods because possible converging results would strengthen our observation in such a particular career group.

**fMRI acquisition parameters**

After morphological examination, participants underwent a second MRI sequence for assessing resting-state functional activity. fMRI exam consisted of 200 volumes of a repeated gradient-echo echo planar imaging sequence for a total imaging time of 6 min and 40 sec (TR/TE: 2000/25 msec; n° axial slices = 39; field of view = 24 mm; thickness/gap = 3 / 0.8 mm; matrix size = 96x96). During the functional scan, we asked the subjects to stay motionless, awake and relaxed with their eyes closed; there were no visual or auditory stimuli present at any time during functional scanning. MRIcron (http://www.cabiatl.com/ mricro/mricron/index.html) was used for converting raw T1 and fMRI DICOM to 4D NIfTI.nii data.

**Resting-state fMRI pre-processing: MELODIC/FSL**

In the first step, we pre-processed functional images using MELODIC tool that is part of the FMRIB’s Software Library. For each subject’s resting-state fMRI dataset, the following preprocessing steps were taken: (i) skull-stripping using the Brain Extraction Tool; (ii) co-registration and normalization of the fMRI dataset to the MNI template brain using FMRIB’s Linear Image Registration Tool; functional images were, then, (iii) denoised with a high-pass temporal filter to remove very low-frequency drifts and physiological high-frequency noise [5,7] and (iv) spatially smoothed with a 8 mm full-width at half maximum spatial filter. (v) Finally, global signal regression was applied.

As resting-state functional connectivity analysis is sensitive to head motion [8], we characterized the head motion as an index representing the peak displacement that was determined by using a previously validated method applied on patients with Parkinson’s disease [4,9]. Specifically, we calculated the Euclidian distance travelled by each subject’s head from the first to the last scan (i.e. combining the movements in the three axes into one value), separately for translation (x, y and z direction) and for rotation (pitch, roll and yaw). Then, a two-sample *t*-test was performed on the peak displacement to examine between group differences in head motion. Overall, the size of head movements during scanning was still on average very small in both groups: translation movements lower than 0.08 mm and rotation movements lower than 0.02 mm (Figure S2). Considering all head’s motion parameters, no significant difference was detected between the two groups (all *t*’s-value > 0.4).


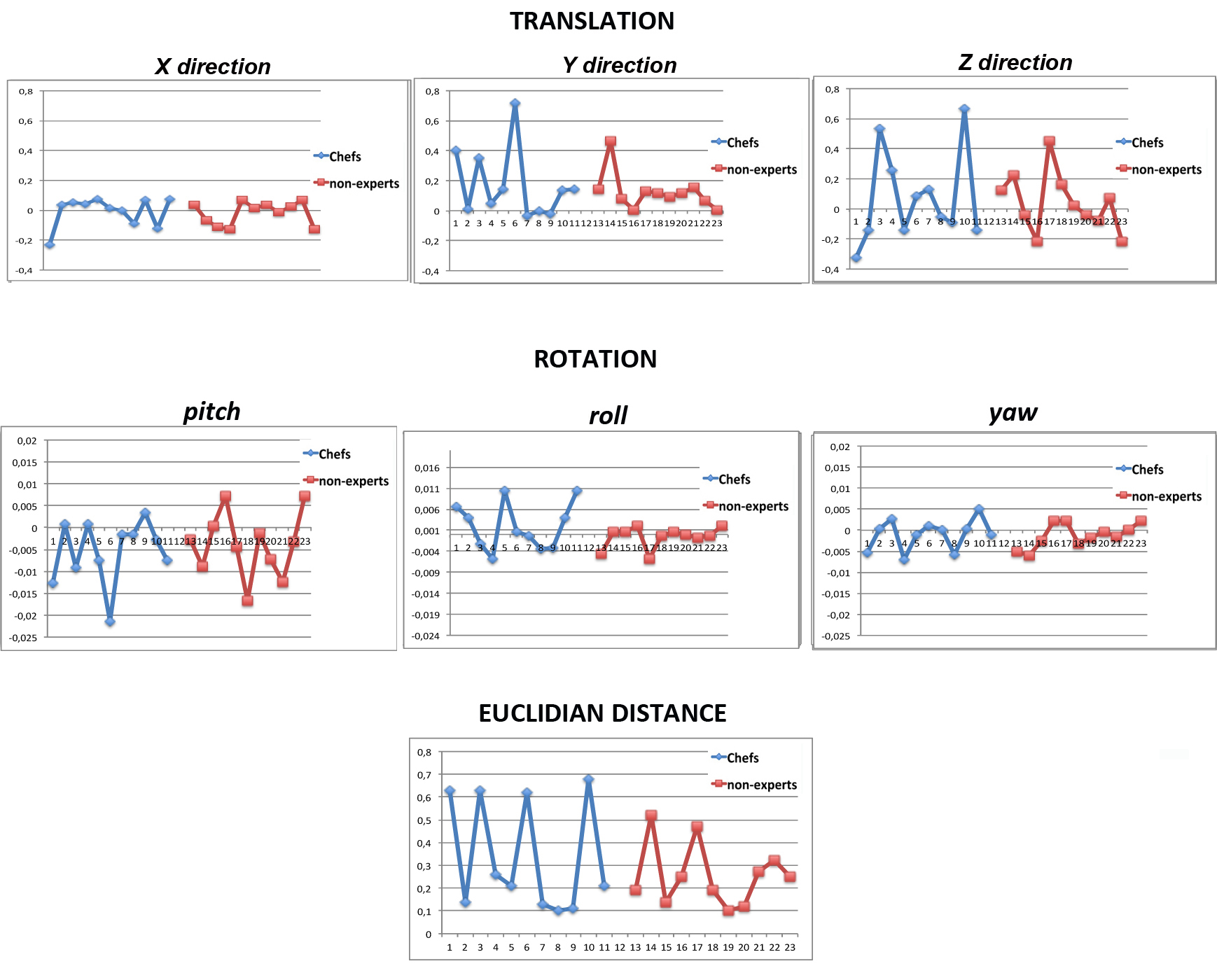


**S2 Fig: Motion artifacts analysis.** Plots of the seven mean motion head parameters during resting-state fMRI session for each single expert and non-expert individuals. Figure shows trend of head motion separately for translation (x, y and z direction, first row) and for rotation (pitch, roll and yaw, second row). In the lower part of the figure we show calculation of the Euclidian distance traveled by each subject’s head from the first to the last scan. No significant motion difference was detected during fMRI measurement.

**Resting-state fMRI pre-processing: CONN toolbox**

Functional data were re-analyzed using the CONN-fMRI functional connectivity toolbox v14 [6], together with Matlab version R2015a and SPM8. This toolbox has been recently validated in several psychological and clinical realms [10-12].

The functional images were first slice-time corrected, realigned to the first scan and registered to the MNI anatomical template image. Next, we evaluated the impact of motion artifacts for each subject. We characterized the head motion using the Artifact Rejection Toolbox (ART; http://www.nitrc.org/projects/artifact_detect/) to create confound regressors (3 translation and 3 rotation parameters) and to remove specific image frames with outliers based on brain activation and head movement. In order to identify outliers in brain activation, the mean global brain activity was calculated as a function of time, and was then Z normalized. The next step consisted in regressing out the nuisance signals involving the head motion parameters, global cerebrospinal fluid and white matter signals. CONN uses the *CompCor* strategy for spatial and temporal preprocessing to define and remove confounds in the BOLD signal to prevent the impact of physiological noise factors and motion in the data [13,14]. This method extracts principal components from white-matter (WM) and cerebrospinal fluid (CSF) time series. WM and CSF voxels are identified via a segmentation of the anatomical images. These components, together with motion parameters derived from ART, are added as confounds in the denoising step of the CONN toolbox [14]. Finally, images were denoised with a band-pass temporal filter (0.008-0.09 Hz) and spatial smoothing using a Gaussian kernel of FWHM 8 mm, since this preprocessing step was found to increase the retest reliability [13,14]. The global signal was excluded.

**Functional connectivity analyses**

**Seed definition**

The MNI coordinates of cerebellar regions showing significant volumetric differences in VBM analysis were employed as seeds for functional connectivity analysis (see Results section in the main document). Two separate 5-mm spheres around these coordinates (anterior and posterior cerebellar seeds) were created with SPM8 and used to perform seed-to-voxel functional connectivity analyses.

**Functional connectivity analyses**

Seed-based analyses were performed separately for the two pre-processing approaches. At the first-level, in both cases, the mean time-series averaged across all voxels within each seed was used as a regression parameter and correlated with all other voxels in the brain in a seed-to-voxel connectivity analyses. At the second-level, an independent two-sample *t*-test analysis was employed to investigate group differences in seed connectivity considering the time courses of the two-cerebellar regions modeled with nuisance parameters.

Functional neuroimaging data were thresholded using two statistical approaches. Firstly, we restricted our statistical analysis to two specific masks of brain regions closely anatomically connected with the anterior and posterior cerebellar “seeds”. These masks were based on established literature identifying regions involved in the motor/cognitive cerebello-cortical pathways [15-17]. Correction for multiple comparisons (family wise error (FWE) < 0.05) within these masks was applied. In particular, for the first cerebellar “motor” seed-based analysis we considered the sensorimotor cortices (BA 3-4) together with basal ganglia and the premotor cortex (BA 6). For the second cerebellar posterior seed, functional reorganization within the cognitive pathway including the prefrontal regions BA 9, 10, 45, 46, as well as the superior parietal cortex (BA 7) was evaluated. All masks were created with the “aal.02” atlas included in the Wake Forest University Pickatlas software version 1.04 (Functional MRI Laboratory at the Wake Forest University School of Medicine; http://www.fmri.wfubmc.edu/download.htm). Each single region was combined into a unique mask, which were in turn used to threshold the second level statistical maps. Second, given the lack of literature data linking brain features in *Chefs*, for exploratory purpose the data were also presented by using a less-stringent, uncorrected threshold (P < 0.001, cluster (k) threshold > 10 voxels) to detect subtle brain differences at a whole-brain level.

**RESULTS**

**Resting-state functional connectivity: MELODIC/FSL**

The comparison between *Chefs* with respect to non-experts revealed significant results (Figure S3). Indeed, the anterior cerebellar lobule showed increased communication with the bilateral secondary somatosensory cortex *(local maxima; t-level= 8.5; P_FWE_= 0.004; cluster (K)= 208; MNI coordinates x: -50; y: -20; z: 20; local maxima; t-level= 6.9; P_FWE_= 0.04; cluster (K)= 48; MNI coordinates x: 52; y: -24; z: 20;* respectively for the left and right sides) and the medial premotor cortex *(t-level= 7.7; P_FWE_= 0.03; cluster (K)= 121; MNI coordinates x: 10; y: 18; z: 48)*. We also found a significant reduction of the functional connectivity between the left Crus II and the right anterior prefrontal cortex *(t-level= 7.83; P_FWE_= 0.03; cluster (K)= 76; MNI coordinates x: 38; y: 54; z: 12).* For exploratory purposes, we also investigated functional changes occurring when a less-stringent uncorrected statistical threshold was considered (P_uncorrected_ < 0.001). No additional data were found.

*
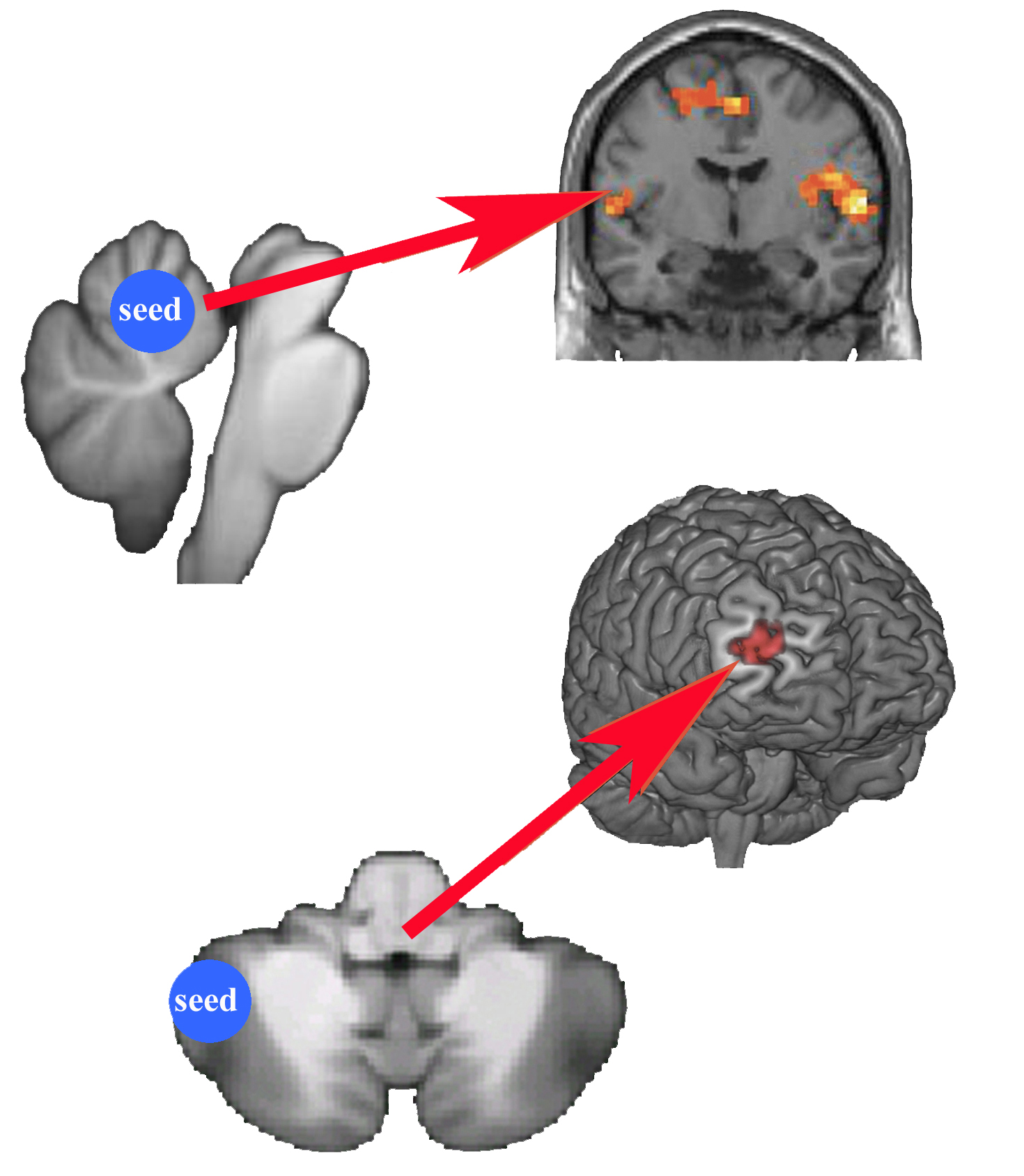
*

**S3 Fig: Seed-based functional connectivity analysis by MELODIC/FSL toolbox.** The comparison between Chefs and non-expert showed increased communication between the seed placed on the anterior cerebellar lobule and the bilateral secondary somatosensory cortex together with the medial premotor cortex. Considering the second seed placed on the posterior cerebellar lobule, Chefs showed decreased connectivity with right anterior prefrontal cortex.

**Resting-state functional connectivity: CONN toolbox**

The comparison between *Chefs* with respect to non-experts did not reveal significant results surviving correction for comparisons. At lower uncorrected statistical threshold, we only found (Figure S4) an increased communication between the seed placed on the anterior vermis and the right motor and premotor cortices *(local maxima; t-level= 4.99; P_uncorrected_<0.001; cluster (K)= 44; MNI coordinates x: 50; y: 0; z: 48*)

*
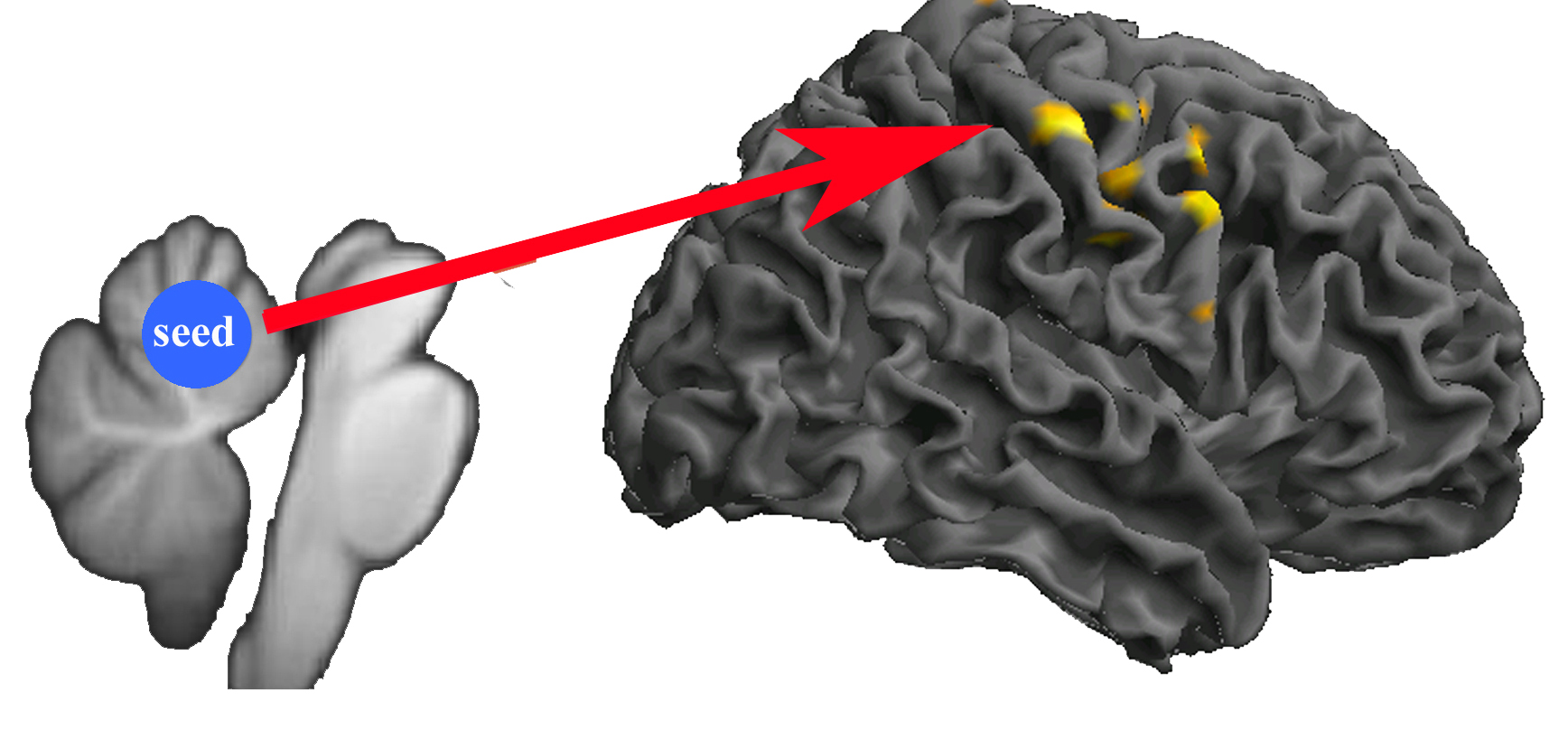
*

**S4 Fig: Seed-based functional connectivity analysis by CONN toolbox.** The comparison between Chefs and non-expert showed increased communication between the seed placed on the anterior cerebellar lobule (left panel) and the right motor and premotor cortices (red blob in right panel), although without reaching significant threshold.

The contrasting findings obtained from the two different resting-state fMRI connectivity approaches did not clarify whether *Chef*s are characterized by evident functional cerebello-cortical reorganization. The only consistent finding would seem the increased communication between the anterior cerebellum and the motor pathway that deserves attention in future studies.

**Additional References**

1. Ashburner J. A fast diffeomorphic image registration algorithm. Neuroimage 2007; 38 (1)**:** 95-113.
2. Laricchiuta D, Petrosini L, Piras F, Macci E, Cutuli D, Chiapponi C, Cerasa A, Picerni E, Caltagirone C, Girardi P, Tamorri SM, Spalletta G. Linking novelty seeking and harm avoidance personality traits to cerebellar volumes. Hum Brain Mapp. 2014; 35(1):285-96.
3. Donzuso G, Cerasa A, Gioia MC, Caracciolo M, Quattrone A. The neuroanatomical correlates of anxiety in a healthy population: differences between the State-Trait Anxiety Inventory and the Hamilton Anxiety Rating Scale. Brain Behav. 2014; 4(4):504-14.
4. Cerasa A, Koch G, Donzuso G, Mangone G, Morelli M, Brusa L, et al. A network centred on the inferior frontal cortex is critically involved in levodopa-induced dyskinesias. Brain 2015; 138(pt 2): 414-427.
5. Beckmann CF, and Smith SM. Probabilistic independent component analysis for functional magnetic resonance imaging. IEEE Trans Med Imaging 2004; 23: 137–152.
6. Whitfield-Gabrieli S, Nieto-Castanon A. Conn: a functional connectivity toolbox for correlated and anticorrelated brain networks. Brain Connect. 2012; 2(3):125-41.
7. Biswal B, Yetkin FZ, Haughton VM, Hyde JS. Functional connectivity in the motor cortex of resting human brain using echo-planar MRI. Magn Reson Med 1995; 34(4): 537-541.
8. Power JD, Schlaggar BL, Petersen SE. Recent progress and outstanding issues in motion correction in resting state fMRI. Neuroimage 2015; 105: 536-551.
9. Wu T, Long X, Wang L, Hallett M, Zang Y, Li K, Chan P. Functional connectivity of cortical motor areas in the resting state in Parkinson’s disease. Hum Brain Mapp 2011; 32(9): 1443-1457.
10. Gupta T, Silverstein SM, Bernard JA, Keane BP, Papathomas TV, Pelletier-Baldelli A, et al. Disruptions in neural connectivity associated with reduced susceptibility to a depth inversion illusion in youth at ultra high risk for psychosis. Neuroimage Clin. 2016; 12:681-690.
11. Beaty RE, Kaufman SB, Benedek M, Jung RE, Kenett YN, Jauk E, et al.. Personality and complex brain networks: The role of openness to experience in default network efficiency. Hum Brain Mapp 2016; 37(2):773-9.
12. Boissoneault J, Letzen J, Lai S, O'Shea A, Craggs J, Robinson ME, Staud R. Abnormal resting state functional connectivity in patients with chronic fatigue syndrome: an arterial spin-labeling fMRI study. Magn Reson Imaging 2016; 34(4):603-8.
13. Shirer WR, Jiang H, Price CM, Ng B, Greicius MD. Optimization of rs-fMRI Pre-processing for Enhanced Signal-Noise Separation, Test-Retest Reliability, and Group Discrimination. Neuroimage. 2015;117: 67-79.
14. Behzadi Y, Restom K, Liau J, Liu TT. A component based noise correction method (CompCor) for BOLD and perfusion based fMRI. Neuroimage. 2007; 37(1):90-101.
15. Bernard JA, Seidler RD, Hassevoort KM, Benson BL, Welsh RC, Wiggins JL, et al. Resting state cortico-cerebellar functional connectivity networks: a comparison of anatomical and self-organizing map approaches. Front Neuroanat. 2012 Aug 10;6:31.
16. Buckner RL, Krienen FM, Castellanos A, Diaz JC, Yeo BT. The organization of the human cerebellum estimated by intrinsic functional connectivity. J Neurophysiol 2011**;** 106(5): 2322-2345.
17. Stoodle CJ, Schmahmann JD. Evidence for topographic organization in the cerebellum of motor control versus cognitive and affective processing. Cortex 2010; 46(7): 831–844.
